# Association of Taxane Type With Patient-Reported Chemotherapy-Induced Peripheral Neuropathy Among Patients With Breast Cancer

**DOI:** 10.1001/jamanetworkopen.2022.39788

**Published:** 2022-11-02

**Authors:** Hongnan Mo, Xiaoyan Yan, Fang Zhao, Yuee Teng, Xiaoying Sun, Zheng Lv, Mengru Cao, Jiuda Zhao, Guohong Song, Bo Pan, Huihui Li, Jingtong Zhai, Binghe Xu, Fei Ma

**Affiliations:** 1 Department of Medical Oncology, National Cancer Center/National Clinical Research Center for Cancer/Cancer Hospital, Chinese Academy of Medical Sciences and Peking Union Medical College, Beijing, China; 2 Department of Biostatistics, Peking University Clinical Research Institute, Beijing, China; 3 Department of Medical Oncology, The First Hospital of China Medical University, Shenyang, China; 4 Department of Medical Oncology, Cancer Hospital of HuanXing ChaoYang District, Beijing, China; 5 Cancer Center, First Affiliated Hospital of Jilin University, Changchun, China; 6 Department of Medical Oncology, Harbin Medical University Cancer Hospital, Harbin, China; 7 Breast Disease Diagnosis and Treatment Center, Affiliated Hospital of Qinghai University & Affiliated Cancer Hospital of Qinghai University, Xining, China; 8 Department of Breast Oncology, Peking University Cancer Hospital and Institute, Beijing, China; 9 Department of Breast Surgery, Peking Union Medical College Hospital, Chinese Academy of Medical Sciences, Beijing, China; 10 Department of Breast Medical Oncology, Shandong Cancer Hospital and Institute, Shandong First Medical University and Shandong Academy of Medical Sciences, Jinan, China

## Abstract

**Importance:**

Understanding the detailed symptom spectrum of chemotherapy-induced peripheral neuropathy (CIPN) could facilitate shared decision-making and promote early intervention.

**Objective:**

To compare the symptom spectrum of patient-reported CIPN associated with nab-paclitaxel, paclitaxel, and docetaxel treatments among patients with breast cancer.

**Design, Setting, and Participants:**

This prospective cohort study was conducted at 9 medical centers across China from 2019 to 2021. Participants included hospitalized women diagnosed with invasive breast cancer, assessed with overlap propensity score weighting. Data were analyzed from from December 2021 to May 2022.

**Exposures:**

Treatment with nab-paclitaxel–, paclitaxel-, or docetaxel-based regimens.

**Main Outcomes and Measures:**

Patient-reported CIPN on the European Organization for Research and Treatment of Cancer Quality of Life Questionnaire: CIPN 20-item instruments, consisting of sensory, motor, and autonomic scales. Multiple regression models were adjusted for baseline patient, tumor, and treatment characteristics.

**Results:**

Of 1234 participants, the mean (SD) age was 50.9 (10.4) years, and 295 patients (23.9%) received nab-paclitaxel, 514 patients (41.7%) received paclitaxel, and 425 patients (34.4%) received docetaxel. The nab-paclitaxel group mostly reported numbness in hands or feet related to sensory symptoms (83 patients [81.4%]), while the paclitaxel and docetaxel groups reported mainly motor (eg, weakness in legs: 60 patients [47.2%] in the paclitaxel group; 52 patients [44.4%] in the docetaxel group) and autonomic (eg, blurred vision: 58 patients [45.7%] in the paclitaxel group; 51 patients [43.6%] in the docetaxel group) symptoms. Patients reported motor symptoms earlier than sensory abnormalities, with a median of 0.4 (95% CI, 0.4-2.3) weeks in the nab-paclitaxel group, 2.7 (95% CI, 1.7-3.4) weeks in the paclitaxel group, and 5.6 (95% CI, 3.1-6.1) weeks in the docetaxel group. After overlap propensity score weighting and compared with the nab-paclitaxel group, the risks of patient-reported CIPN were lower in the paclitaxel (hazard ratio [HR], 0.59 [95% CI, 0.41-0.87]; *P* = .008) and the docetaxel (HR, 0.65 [95% CI, 0.45-0.94]; *P* = .02) groups. Similarly, patients who received paclitaxel (HR, 0.44 [95% CI, 0.30-0.64]; *P* < .001) or docetaxel (HR, 0.52 [95% CI, 0.36-0.75]; *P* < .001) reported less sensory discomfort compared with those who received nab-paclitaxel. However, the risk of patients in the paclitaxel or docetaxel groups reporting motor (paclitaxel: HR, 0.76 [95% CI, 0.52-1.11]; *P* = .15; docetaxel: HR, 0.69 [95% CI, 0.47-1.01]; *P* = .05) and/or autonomic (paclitaxel: HR, 1.00 [95% CI, 0.68-1.49]; *P* = .98; docetaxel: HR, 0.88 [95% CI, 0.59-1.30]; *P* = .52) symptoms was not lower than that in the nab-paclitaxel group.

**Conclusions and Relevance:**

In this cohort study of women with invasive breast cancer, nab-paclitaxel was associated with more severe CIPN than either paclitaxel or docetaxel. In addition to sensory symptoms, the risk of motor and autonomic abnormalities was not low among these 3 taxanes, and patients-reported motor symptoms even earlier than sensory symptoms. These findings may facilitate early detection and intervention for CIPN in taxane treatments for breast cancer.

## Key Points

**Question:**

What is the symptom spectrum of taxane-related neurotoxic effects in patients with breast cancer?

**Findings:**

In this cohort study involving 1234 women, patients treated with nab-paclitaxel mostly reported numbness in their hands or feet related to sensory conditions, while patients in the paclitaxel and docetaxel groups reported mainly motor and autonomic symptoms. After overlap propensity score weighting, the risks of patient-reported neurotoxic effects in paclitaxel and docetaxel groups were significantly lower than in the nab-paclitaxel group.

**Meaning:**

These findings may facilitate early detection and intervention of taxane-related neurotoxic effects in patients with breast cancer.

## Teaser

This cohort study compares the symptom spectrum of patient-reported chemotherapy-induced peripheral neuropathy associated with nab-paclitaxel, paclitaxel, and docetaxel treatments among patients with breast cancer.

## Introduction

Breast cancer has become the most common malignant neoplasm in China and globally.^1,2^ Taxanes are one of the most important treatment drugs for breast cancer. They are widely used in adjuvant therapy for patients with early-stage cancer, neoadjuvant therapy for patients with locally advanced disease, and palliative therapy for patients with advanced disease. Chemotherapy-induced peripheral neuropathy (CIPN) has been recognized as a common adverse event of taxanes, exerting a negative impact on patients’ quality of life and even clinical outcomes. Approximately 60% to 70% patients receiving taxanes experience CIPN, which has become one of the main reasons for early termination of treatment.^3,4^ Symptoms of taxane-related neuropathy typically occur during the first 2 months, progress during treatment, and may even worsen after cessation of therapy.^5^ With increasing numbers of patients surviving breast cancer owing to improved antitumor treatments, CIPN caused by taxanes has become a treatment-related adverse effect that oncologists must focus on. There are emerging therapeutics that can be explored clinically to improve the management of this debilitating toxic effect.^4^ Therefore, we must have a clear and comprehensive understanding of the clinical manifestations of taxane-related CIPN to avoid delayed detection and treatment of this adverse effect.

Currently available grading systems for CIPN, including the National Cancer Institute Common Toxicity Criteria for Adverse Events (NCI CTCAE) and the Total Neuropathy Score clinical version,^6^ typically use a combination of clinical and paraclinical parameters that mainly rely on the judgment of clinical practitioners. However, discordance has been found between patient-reported symptoms and their documentation in the medical record by clinicians.^7^ Clinical benefits, even improved survival, were associated with patient-reported symptom monitoring in patients with metastatic cancer.^8,9^ So, it is logical that CIPN assessment should, at least partly, be based on patient-reported data. In the past, taxane-related CIPN was commonly known as a condition affecting peripheral sensory nerves and mainly included tingling, numbness, and pain of the distal extremities.^3^ Thus, traditional questionnaires for evaluating chemotherapy-related neurotoxic effects, represented by the Functional Assessment of Cancer Treatment/Gynecologic Oncology Group–Neurotoxicity (FACT/GOG-Ntx), were often limited to the evaluation of sensory system symptoms while ignoring other clinical manifestations of neuropathy, such as the motor and autonomic systems. Well-validated neuropathy measurement tools, such as the European Organization for Research and Treatment of Cancer, Quality of Life Questionnaire, Chemotherapy-Induced Peripheral Neuropathy 20-item instruments (EORTC QLQ-CIPN20), have been recently used in several clinical studies.^10,11^ The data from these studies allows the recording of more detailed neuropathy clinical manifestations caused by taxanes. Detailed phenotyping of the clinical syndrome of taxane-related CIPN contributes to the individualized regimen selection and prompt management of adverse events in patients with breast cancer.

Many kinds of taxanes are clinically available, including paclitaxel, docetaxel, and nab-paclitaxel. In previous clinical trials with efficacy as the primary end point, clinicians assessed taxane-related neurotoxic effects mainly by NCI CTCAE and found that the incidence of nab-paclitaxel–related neurotoxic effects was higher than that of paclitaxel,^12^ while there was no significant difference between paclitaxel and docetaxel.^13^ Recently, several studies focusing on taxane-related CIPN have described the acute symptoms and chronic manifestations of CIPN in patients with breast cancer treated with diverse taxanes.^11,14,15,16,17^ However, most of the studies only focused on sensory system symptoms as the main manifestations of taxane-related CIPN. Moreover, these studies used diverse taxanes and inconsistent evaluation scales in patients with different treatment stages, so their results are difficult to compare directly. The purpose of this study is to compare CIPN among different taxanes in a large study cohort using the EORTC QLQ-CIPN20 and FACT/GOG-Ntx questionnaires for patient-reported CIPN evaluation.

## Methods

This cohort study was approved from the institutional review boards of the National Cancer Center/Cancer Hospital, Chinese Academy of Medical Sciences (coordinating center) and each participating site. Written informed consent was obtained from each participant. This study followed the Strengthening the Reporting of Observational Studies in Epidemiology (STROBE) reporting guideline.

### Study Population

This prospective cohort study recruited women from 9 medical centers across China from August 1, 2019, to August 31, 2021, namely the National Cancer Center/Cancer Hospital, Chinese Academy of Medical Sciences; Peking Union Medical College Hospital; Beijing Cancer Hospital; Shandong Cancer Hospital; Heilongjiang Cancer Hospital; The First Hospital of China Medical Hospital; The First Bethune Hospital of Jilin University; The First Affiliated Hospital of Qinghai University; and Cancer Hospital of Huanxing. Included women were aged 18 years or older at the time of diagnoses, were diagnosed with invasive breast cancer based on pathology findings, and were planning or undergoing chemotherapy with a taxane-containing regimen at these centers. The exclusion criteria included concomitant use or history of platinum, no taxanes used, and no questionnaires completed after taxane treatment. The attending physicians at each site decided on the chemotherapy regimens based on common clinical guidelines and the individual situation of each patient.

### Data Collection

Baseline is defined as the date of initiation of antineoplastic treatment, which is the first day of the first cycle of taxane therapy. A survey consisting of 2 well-established questionnaires, the EORTC QLQ-CIPN20 and FACT/GOG-Ntx, was used to evaluate patient-reported CIPN at the baseline and/or at the patient’s visit to the medical center after the end of each taxane cycle. Patients were prompted to complete scores at each visit either filling out the paper questionnaires or using a mobile phone in the waiting room.

The validated EORTC QLQ-CIPN20 is a 20-item instrument providing valuable information on CIPN-related symptoms and functional limitations of patients, with 9 sensory items, 8 motor items and 3 autonomic items,^18^ with each rated on a 4-point scale, with 1 indicating not at all and 4, very much. A higher score is equivalent to more or worse symptoms. The validated FACT/GOG-Ntx is an 11-item subscale that evaluates symptoms associated with CIPN on a 5-point scale, from 0 indicating not at all to 4, very much.^19^ The Chinese Mandarin versions of both scales were obtained from the official websites through authorization. Surveys captured patient-reported age and ethnicity. Ethnicity was dichotomized as Han and other minority grous (including Bai, Hui, Kazakh, Korean, Manchu, Mongolian, Russian, Tibetan, Tujia, Xibo, Yi, and Zhuang) and was included because disease characteristics and treatment response may vary by ethnicity. An electronic health record review, including clinical and treatment information, was conducted in November 2021.

The primary outcome of interest was the incidence of patient-reported CIPN assessed by EORTC QLQ-CIPN20 scores. Patient-reported neurotoxic effects were considered to occur when any of the EORTC QLQ-CIPN20 item scores exceeded 1. Left-censored was defined as when CIPN occurred before the first questionnaire, interval censored was when CIPN occurred between 2 questionnaires, and right-censored was when there was no CIPN by the last questionnaire. Secondary outcomes included scores on the sensory, motor, and autonomic scales of the EORTC QLQ-CIPN20, as well as the FACT/GOG-Ntx scores.

The primary independent variable was the types of taxanes used (ie, nab-paclitaxel, paclitaxel, or docetaxel). Taxane dosage and duration are determined by the physician according to the guideline recommendations and the patient’s condition. Demographic and clinical variables included age, ethnicity (Han vs ethnic minority), body mass index (BMI), diabetes status, history of peripheral neuropathy, treatment stage (ie, neoadjuvant, adjuvant, or palliative), chemotherapy regimen (ie, monotherapy or combination), and number of chemotherapy cycles.

### Statistical Analysis

Baseline characteristics were compared across taxane types using analysis of variance (ANOVA) for continuous variables and χ^2^ tests for categorical variables. Additionally, the Cohen κ statistic was used to measure interrater reliability between questionnaires.

To compare group differences in EORTC QLQ-CIPN20 scores, linear mixed-effects models were used, and subsequent pairwise comparisons were adjusted using the Scheffé method. Exposure factors, time, and their interaction effects were included in the model. Since this was a secondary outcome, we did not adjust for other covariates in this model. The incidence rate of patient-reported CIPN was estimated using the expectation-maximization and iterative convex minorant algorithms, a generalization of the Kaplan-Meier curves for interval-censored data.^20^ The incidence rates of each group were compared using the generalized log-rank test, and the Tukey method was used for pairwise comparisons following the overall comparison.

To estimate the association between the type of taxanes and the incidence of patient-reported CIPN, we used semiparametric proportional hazards regression models,^21^ since our data are subject to left-censored, right-censored, and interval-censored observations. Three regression models were used: the first model only included the type of taxane; the second model further added patient age, ethnicity, BMI, diabetes, history of peripheral neuropathy, treatment stage, and chemotherapy regimens; and the third model extended the second model by including cycles of prior taxane treatment and Ki-67. Using these models, we estimated hazard ratios (HRs) and 95% CIs reflecting the independent association of each factor with patient-reported CIPN.

In addition, to account for the nonrandomized treatment administration of taxane types, 3 propensity score–matching methods were used to reduce the confounding effects. A generalized propensity score for the type of taxane used was estimated from a multinomial logistic regression model containing patient age, ethnicity, BMI, diabetes, history of peripheral neuropathy, treatment stage, and chemotherapy regimens.

The primary analysis used generalized overlap weighting,^22^ and pairwise absolute standardized differences (ASD) were used for covariate balance assessment. When the absolute value of ASD is less than 0.1, the balance is considered to be good. Subsequently, overlap propensity score–weighted semiparametric proportional hazards models were used to investigate associations between the type of taxanes used and the probability of patient-reported CIPN as measured by EORTC QLQ-CIPN20 scores as well as other clinical outcomes.

We conducted a secondary analysis that used inverse probability weighting^23^ and another that included the propensity score as an additional covariate.^24^ In the inverse-probability–weighted analysis, the individual propensities for the types of taxanes were estimated using a generalized boosted model that included the same aforementioned covariates. Semiparametric proportional hazards models that used the inverse-probability weights were reported. In the covariate adjustment, the 2 propensity scores corresponding to the use of paclitaxel and docetaxel were included as covariates in the regression model of the outcome variable. Missing values were listed separately and treated as an individual category. Two-sided *P* < .05 was considered statistically significant. All analyses were performed using SAS software version 9.4 (SAS Institute) and R software version 4.2.0 (R Project for Statistical Computing). Data analysis was conducted from December 2021 to May 2022.

## Results

### Cohort Characteristics

**Figure 1.**
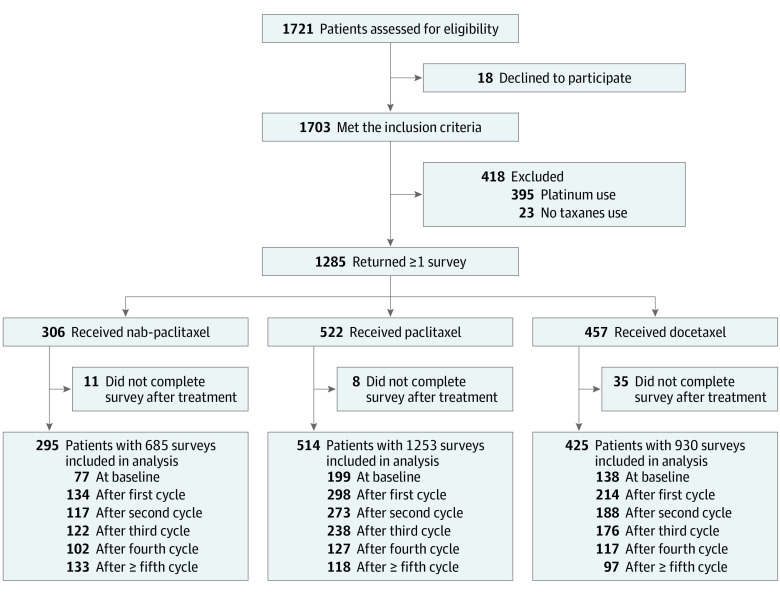
Participant Selection Flowchart

A total of 1234 patients with breast cancer who received taxane-containing chemotherapy were included in the analysis (Figure 1). Participants were all women, by design, and comprised 13 ethnic groups from 30 provinces of mainland China. Their overall mean (SD) age was 50.9 (10.4) years. The baseline demographic and clinical characteristics of the cohort prior to propensity score weighting are summarized in Table 1. Of the full cohort, 295 patients (23.9%) received nab-paclitaxel, 514 patients (41.7%) received paclitaxel, and 425 patients (34.4%) received docetaxel. The geographic region, history of diabetes, history of peripheral neuropathy, Ki-67, treatment stage, and chemotherapy regimens were significantly different among these 3 groups (Table 1).

**Table 1.** Demographic and Clinical Characteristics of the Cohort According to the Type of Taxanes

Characteristic	Patients, No. (%)				*P* value
	Nab-paclitaxel (n = 295)	Paclitaxel (n = 514)	Docetaxel (n = 425)	Overall (N = 1234)	
Age, mean (SD), y	51.9 (11.1)	50.6 (10.5)	50.7 (9.6)	50.9 (10.4)	.20
Ethnicity					
Han	276 (93.6)	484 (94.2)	395 (92.9)	1155 (93.6)	.75
Other minority group^a^	19 (6.4)	30 (5.8)	30 (7.1)	79 (6.4)	
Geographic region					
Central China	13 (4.4)	30 (5.8)	12 (2.8)	55 (4.5)	<.001
East China	24 (8.1)	62 (12.1)	34 (8.0)	120 (9.7)	
North China	121 (41.0)	327 (63.6)	203 (47.8)	651 (52.8)	
Northeast China	122 (41.4)	73 (14.2)	161 (37.9)	356 (28.8)	
Northwest China	9 (3.1)	7 (1.4)	8 (1.9)	24 (1.9)	
South China	5 (1.7)	6 (1.2)	4 (0.9)	15 (1.2)	
Southwest China	1 (0.3)	9 (1.7)	3 (0.7)	13 (1.1)	
BMI, mean (SD)	24.1 (3.2)	23.8 (3.4)	24.0 (3.6)	23.9 (3.4)	.59
Diabetes					
No	262 (88.8)	487 (94.7)	406 (95.5)	1155 (93.6)	<.001
Yes	33 (11.2)	27 (5.3)	19 (4.5)	79 (6.4)	
History of peripheral neuropathy					
No	286 (96.9)	510 (99.2)	413 (97.2)	1209 (98.0)	.03
Yes	9 (3.1)	4 (0.8)	12 (2.8)	25 (2.0)	
Tumor Ki-67, %					
<20	26 (8.8)	100 (19.5)	75 (17.7)	201 (16.3)	<.001
≥20	176 (59.7)	412 (80.1)	231 (54.3)	819 (66.4)	
Missing	93 (31.5)	2 (0.4)	119 (28.0)	214 (17.3)	
Treatment stage					
Adjuvant	112 (38.0)	400 (77.8)	328 (77.2)	840 (68.1)	<.001
Neoadjuvant	46 (15.6)	84 (16.3)	57 (13.4)	187 (15.1)	
Palliative	137 (46.4)	30 (5.8)	40 (9.4)	207 (16.8)	
Chemotherapy regimens					
Combinations^b^	172 (58.3)	249 (48.4)	325 (76.5)	746 (60.5)	<.001
Monotherapy	123 (41.7)	265 (51.6)	100 (23.5)	488 (39.5)	

Abbreviation: BMI, body mass index (calculated as weight in kilograms divided by height in meters squared).

^a^ Including 12 ethnic minority groups: Bai, Hui, Kazakh, Korean, Manchu, Mongolian, Russian, Tibetan, Tujia, Xibo, Yi, and Zhuang.

^b^ Taxanes in combination with other chemotherapeutic drugs.

A total of 2868 surveys were completed; CIPN was reported by patients in 2205 EORTC QLQ-CIPN20 questionnaires (76.9%) and in 2274 FACT/GOG-Ntx scores questionnaires (79.3%). Patient-reported CIPN assessed by these questionnaires had substantial agreement (κ = 0.70 [95% CI, 0.67-0.73]; *P* < .001).

### Patient-Reported CIPN After Use of Taxanes

**Figure 2.**
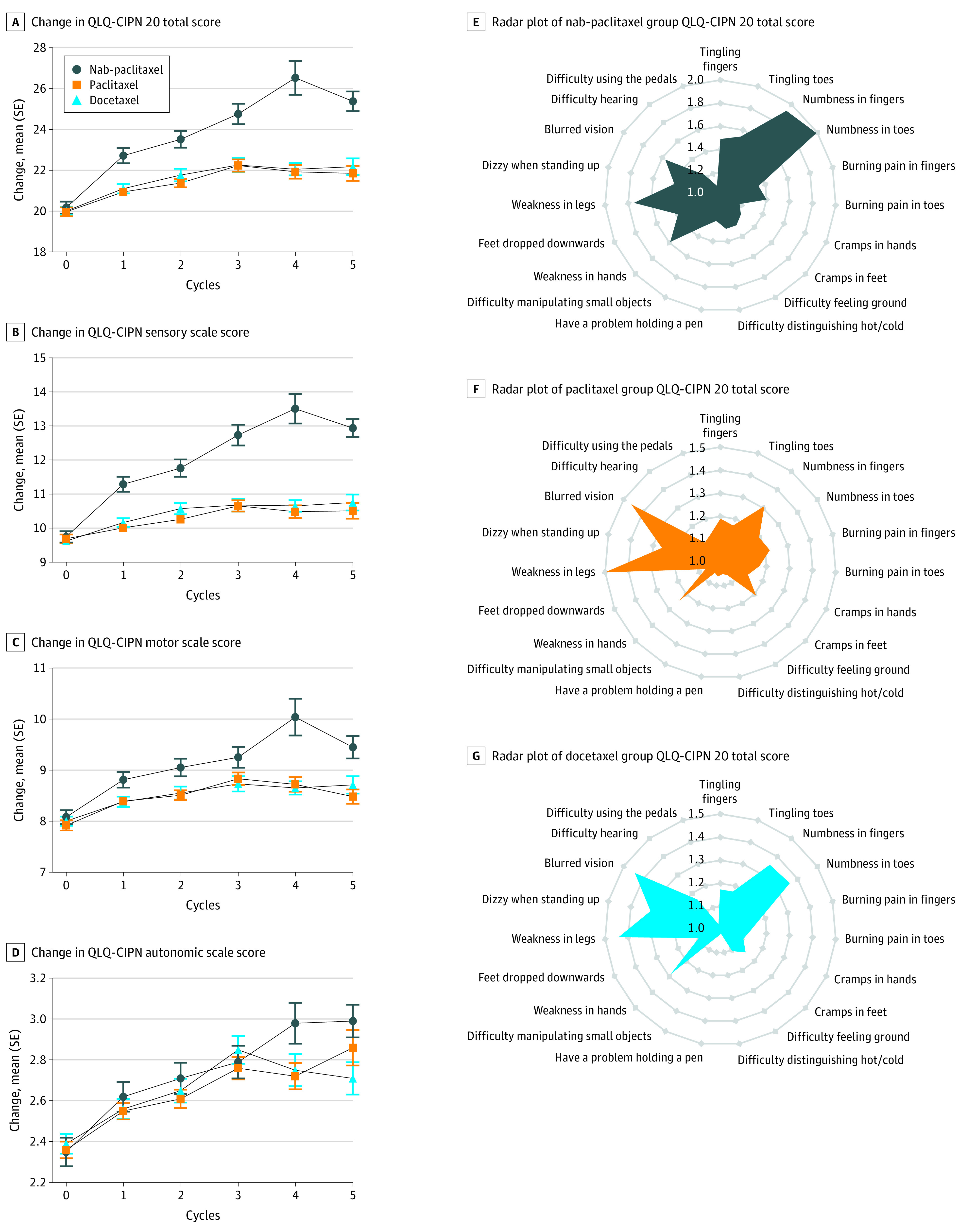
Association Between Types of Taxane and Patient-Reported Neurotoxic Effects A-D, Dynamic changes in European Organization for Research and Treatment of Cancer Quality of Life Questionnaire: Chemotherapy-Induced Peripheral Neuropathy 20-item (QLQ-CIPN20) scores E-G, Radar plots of mean scores in 19 items in the QLQ-CIPN 20 instruments. Since patients included in this cohort were all women and the 20th question only needs to be answered by men, this item is not applicable.

Results of ANOVA based on linear mixed-effects models showed that, after taxane treatment, the EORTC QLQ-CIPN20 total scores of patients in the paclitaxel and docetaxel groups were lower than those in the nab-paclitaxel group (Figure 2A; eTable 1 in the Supplement). The paclitaxel and docetaxel groups had significantly lower sensory scores (Figure 2B) than the nab-paclitaxel group, but the motor and autonomic scores were similar (Figure 2C and 2D; eTable 1 in the Supplement). After 4 cycles of taxane treatment, patients who received different types of taxanes reported a diverse spectrum of CIPN (Figure 2E). The most frequently reported symptoms of patients in the nab-paclitaxel group were numbness in hands or feet related to sensory symptoms (83 patients [81.4%]), while motor symptoms, such as weakness in legs, were also reported. Conversely, the most frequently reported symptoms in patients in the paclitaxel group were motor (eg, weakness in legs: 60 patients [47.2%]) and autonomic (eg, blurred vision: 58 patients [45.7%]) symptoms, while sensory symptoms were relatively mild (numbness in hands). Patients in the docetaxel group reported neurotoxic symptoms similar to those in the paclitaxel group, mainly motor (eg, weakness in legs: 52 patients [44.4%]) and autonomic (eg, blurred vision: 51 patients [43.6%]) symptoms, with relatively mild sensory symptoms (numbness in hands or feet).

**Figure 3.**
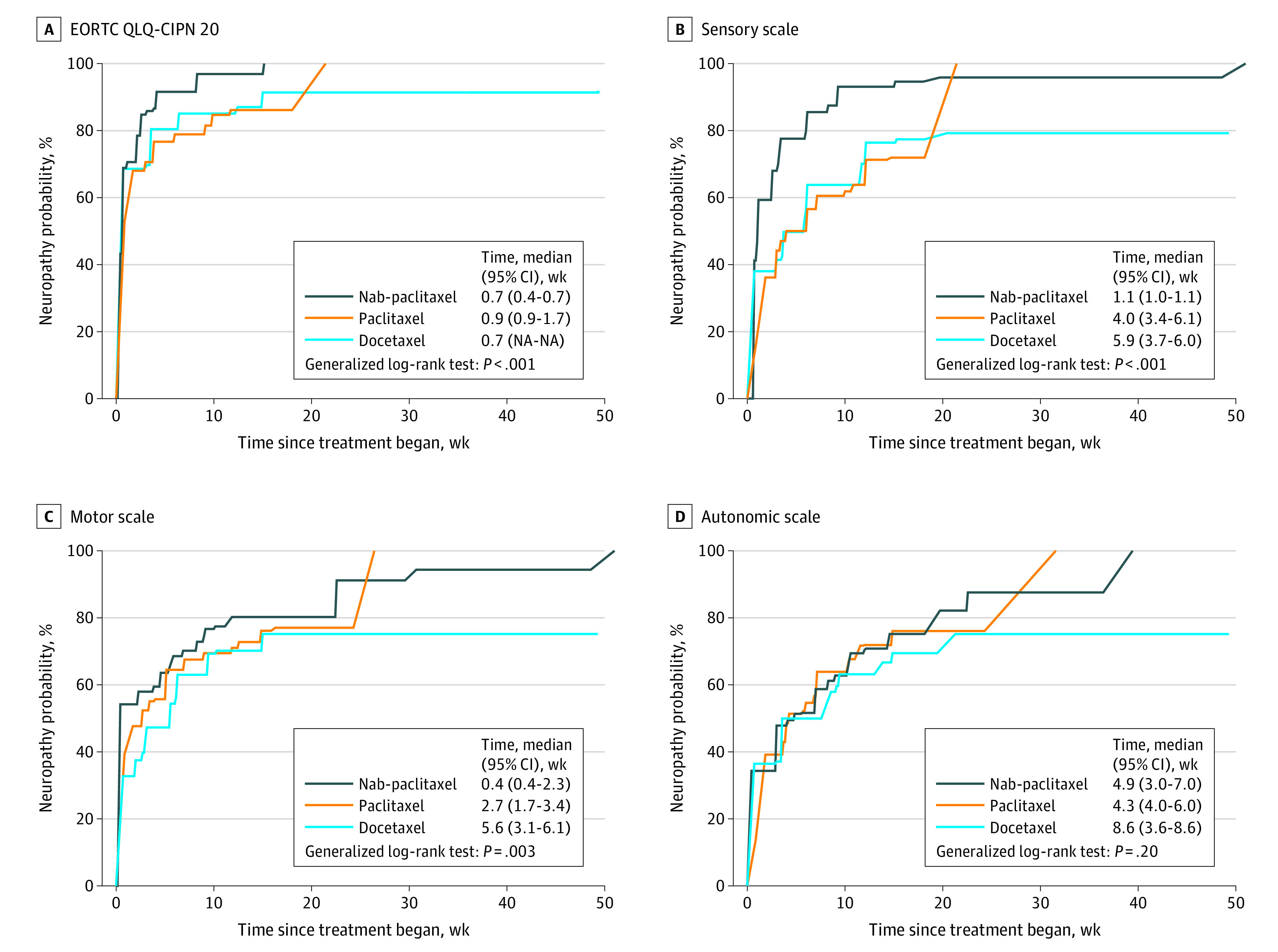
Incidence of Patient-Reported Neuropathy Estimated by the Expectation-Maximization and Iterative Convex Minorant Algorithms Neuropathy was evaluated by the European Organization for Research and Treatment of Cancer Quality of Life Questionnaire: Chemotherapy-Induced Peripheral Neuropathy 20-item (EORTC QLQ-CIPN20) instruments. Baseline is defined as the date of initiation of treatment.

At 6 weeks after initiation of treatment, CIPN was most frequently reported by patients in the nab-paclitaxel group (91.6%; *P* < .001); there was no significant difference between patients in the paclitaxel and docetaxel groups (78.9% vs 80.4%; *P* = .61) (Figure 3A). In the sensory scales, the median time to patient-reported sensory symptoms was 1.1 (95% CI, 1.0-1.1) weeks after initiation of treatment in the nab-paclitaxel group, but later in the paclitaxel (4.0 [95% CI, 3.4-6.1] weeks) and docetaxel groups (5.9 [95% CI, 3.7-6.0] weeks) (Figure 3B). Notably, patients reported motor symptoms earlier than sensory symptoms, with a median reporting time of 0.4 (95% CI, 0.4-2.3) weeks in the nab-paclitaxel group, 2.7 (95% CI, 1.7-3.4) weeks in the paclitaxel group, and 5.6 (95% CI, 3.1-6.1) weeks in the docetaxel group (Figure 3C). As for autonomic symptoms, there were no significant differences among the 3 groups, with the median patient-reported time being 4.9 (95% CI, 3.0-7.0) weeks in the nab-paclitaxel group, 4.3 (95% CI, 4.0-6.0) weeks in the paclitaxel group, and 8.6 (95% CI, 3.6-8.6) weeks in the docetaxel group (Figure 3D).

The results of multivariate analysis suggested that the type of taxane, as well as previous treatment cycles and treatment stage, were independent factors associated with patient-reported CIPN (eTable 2 and eTable 3 in the Supplement). Compared with patients treated with nab-paclitaxel, those treated with paclitaxel (HR, 0.61 [95% CI, 0.49-0.75]; *P* < .001) or docetaxel (HR, 0.66 [95% CI, 0.54-0.82]; *P* < .001) had lower risks of CIPN. Likewise, on sensory and motor scales, patients who received paclitaxel or docetaxel were less likely to report discomfort than those who had received nab-paclitaxel. The likelihood of patients reporting autonomic symptoms was essentially the same among the 3 groups (eTable 2 in the Supplement).

Propensity score distributions according to the types of taxanes used are shown in eFigure 1 in the Supplement. After overlap propensity score weighting (eTable 4 and eFigure 2 in the Supplement), the risks of patient-reported CIPN in the paclitaxel group (HR, 0.59 [95% CI, 0.41-0.87]; *P* = .008) or the docetaxel group (HR, 0.65 [95% CI, 0.45-0.94]; *P* = .02) were lower compared with the nab-paclitaxel group (Table 2; eTable 5 in the Supplement). Similarly, in terms of sensory scales, patients who received paclitaxel (HR, 0.44 [95% CI, 0.30-0.64]; *P* < .001) or docetaxel (HR, 0.52 [95% CI, 0.36-0.75]; *P* < .001) reported less sensory discomfort compared with those who received nab-paclitaxel. However, the risk of patients in the paclitaxel or docetaxel group reporting motor symptoms (paclitaxel: HR, 0.76 [95% CI, 0.52-1.11]; *P* = .15; docetaxel: HR, 0.69 [95% CI, 0.47-1.01]; *P* = .05) was not lower than that of those in the nab-paclitaxel group; the same was true for autonomic symptoms (paclitaxel: HR, 1.00 [95% CI, 0.68-1.49]; *P* = .98; docetaxel: HR, 0.88 [95% CI, 0.59-1.30]; *P* = .52) (Table 2).

**Table 2.** Overlap-Weighted Pairwise Association Between Types of Taxanes and Patient-Reported Chemotherapy-Induced Peripheral Neuropathy Among Patients With Breast Cancer

EORTC QLQ-CIPN20 score	HR (95% CI)^a^	*P* value	HR (95% CI)^b^	*P* value	HR (95% CI)^c^	*P* value
Total Score						
Paclitaxel vs nab-paclitaxel	0.68 (0.48-0.97)	.03	0.67 (0.47-0.96)	.03	0.59 (0.41-0.87)	.01
Docetaxel vs nab-paclitaxel	0.66 (0.46-0.95)	.02	0.66 (0.46-0.95)	.03	0.65 (0.45-0.94)	.02
Docetaxel vs paclitaxel	0.97 (0.69-1.36)	.85	0.98 (0.69-1.39)	.91	1.09 (0.75-1.59)	.65
Sensory scale						
Paclitaxel vs nab-paclitaxel	0.49 (0.34-0.70)	<.001	0.48 (0.33-0.68)	<.001	0.44 (0.30-0.64)	<.001
Docetaxel vs nab-paclitaxel	0.54 (0.38-0.77)	<.001	0.53 (0.37-0.76)	<.001	0.52 (0.36-0.75)	<.001
Docetaxel vs paclitaxel	1.10 (0.76-1.58)	.61	1.12 (0.77-1.62)	.55	1.19 (0.81-1.77)	.38
Motor scale						
Paclitaxel vs nab-paclitaxel	0.93 (0.65-1.34)	.71	0.91 (0.64-1.31)	.63	0.76 (0.52-1.11)	.15
Docetaxel vs nab-paclitaxel	0.71 (0.49-1.02)	.06	0.71 (0.49-1.03)	.07	0.69 (0.47-1.01)	.05
Docetaxel vs paclitaxel	0.75 (0.52-1.09)	.13	0.77 (0.53-1.12)	.17	0.91 (0.62-1.35)	.65
Autonomic scale						
Paclitaxel vs nab-paclitaxel	1.10 (0.76-1.60)	.61	1.08 (0.74-1.57)	.68	1.00 (0.68-1.49)	.98
Docetaxel vs nab-paclitaxel	0.85 (0.58-1.25)	.41	0.86 (0.58-1.26)	.43	0.88 (0.59-1.30)	.52
Docetaxel vs paclitaxel	0.77 (0.53-1.13)	.18	0.79 (0.54-1.16)	.23	0.88 (0.58-1.32)	.53

Abbreviations: EORTC QLQ-CIPN20, European Organization for Research and Treatment of Cancer Quality of Life Questionnaire: Chemotherapy-Induced Peripheral Neuropathy 20-item; HR, hazard ratio.

^a^ Univariate model only include types of taxanes.

^b^ Multivariable models accounted for baseline characteristics, including patient age, ethnicity, body mass index, diabetes, history of peripheral neuropathy, treatment stage, and chemotherapy regimens.

^c^ Model further adjusted for treatment cycles and Ki-67.

### Sensitivity Analysis

In the sensitivity analysis with inverse probability weighting according to the propensity score, patients in the paclitaxel group (HR, 0.60 [95% CI, 0.50-0.73]; *P* < .001) and the docetaxel group (HR, 0.66 [95% CI, 0.57-0.76]; *P* < .001) were less likely to report CIPN than those in the nab-paclitaxel group (eTable 6 in the Supplement). Covariate adjustment using propensity scores was also used and yielded similar results. The risk of patient-reported CIPN was significantly reduced with paclitaxel (HR, 0.69 [95% CI, 0.56-0.84]; *P* < .001) or docetaxel (HR, 0.69 [95% CI, 0.56-0.85]; *P* < .001) compared with nab-paclitaxel.

## Discussion

In this prospective, multicenter cohort study of women with breast cancer, we found that patients who received different types of taxanes reported different spectrums of CIPN. The most frequently reported symptoms by patients in the nab-paclitaxel group were numbness in hands or feet related to sensory symptoms, while patients in the paclitaxel and docetaxel groups reported mainly motor (weakness in legs) and autonomic (blurred vision) symptoms. Additionally, the risk of motor and autonomic symptoms was not low among these 3 taxanes, and patients reported motor symptoms even earlier than sensory symptoms. After controlling for patient- and tumor-related factors, such as treatment stage and cycles, docetaxel and paclitaxel were associated with a lower risk of patient-reported CIPN compared with nab-paclitaxel.

To our knowledge, our study is the first to suggest that different taxanes have different neurotoxic profiles in terms of patient-reported outcomes. While the most common manifestation of taxane-related CIPN is numbness of the hands and feet, motor- and autonomic-related adverse events are not uncommon after taxane therapy, with symptoms appearing even before sensory abnormalities. Indeed, patients in the paclitaxel and docetaxel groups reported mainly motor (weakness in legs) and autonomic (blurred vision) symptoms. That might be due to steroids used as premedication, since steroid-associated myopathy is the most frequent cause of muscle disorders in patients with cancer.^25,26^ Most of the previous clinical studies on taxane-related neurotoxic effects have focused on sensory symptoms,^12,27,28^ and insufficient attention has been paid to the manifestations of motor and autonomic symptoms. This may delay the detection and treatment of neurotoxic effects and should be of concern to clinical practitioners.

With the gradual prolonging of survival in patients with breast cancer, increasing attention has been paid to the quality of life of patients. Previous studies evaluating CIPN have often relied on the judgment of the investigator.^29,30,31^ In recent years, patient-reported outcomes have been increasingly valued and have been included as study end points in several clinical trials.^11,32,33,34^ A study by Reshma et al^35^ compared patient-reported outcomes and clinician-assessed adverse events in patients receiving radiotherapy and found significant differences between them. Since clinicians tend to underestimate CIPN symptoms experienced by patients,^36^ collecting patient-reported outcomes related to CIPN may be essential for taxane-related trials. To date, the most widely used questionnaires for evaluating patient-reported CIPN are the EORTC QLQ-CIPN20 and FACT/GOG-Ntx questionnaires. Our findings suggest that the traditional FACT/GOG-Ntx questionnaire is in good agreement with the EORTC QLQ-CIPN20 questionnaire in the detection of CIPN. Both questionnaires can be used clinically to help physicians and patients discover taxane-related CIPN.

### Limitations

There are several limitations to this study. First, this study is an observational cohort study and not a randomized trial, so there may be unmeasured confounders. Second, a percentage of patients did not complete the questionnaire at each cycle of treatment as planned. Missing observations may cause attrition bias. Third, the analysis did not adjust for the educational level of the patients, which may influence outcomes. These limitations notwithstanding, this study is bolstered by the use of various statistical methods, such as semiparametric proportional hazards models and multiple regression models with propensity score–based overlapping weighting.

## Conclusions

The findings of this cohort study suggest that patients receiving different types of taxanes may exhibit diverse CIPN profiles. Nab-paclitaxel was associated with higher scores and a higher incidence of CIPN than either paclitaxel or docetaxel. In addition to sensory symptoms, the risks of patient-reported motor and autonomic symptoms were not low among these 3 taxanes. These findings may facilitate early detection and improve the management of taxane-related CIPN for patients with breast cancer.

## Notes

Supplement. **eTable 1.** The EORTC QLQ-CIPN20 Scores Before and After Taxane Treatment **eTable 2.** Pairwise Association Between Types of Taxanes and Patient-Reported CIPN Among Patients With Breast Cancer **eTable 3.** Multivariate Analysis Using Semiparametric Proportional Hazards Models **eFigure 1.** Propensity Score Distributions According to the Types of Taxanes Used **eTable 4.** Overlap Propensity Score–Weighted Demographic and Clinical Characteristics of the Cohort **eFigure 2.** Covariate Balance Assessment in Overlap Weighting Using Maximum Pairwise Absolute Standardized Difference (ASD) **eTable 5.** Overlap Propensity Score–Weighted Multivariate Analysis **eTable 6.** Pairwise Association Between Types of Taxanes and Patient-Reported CIPN in the Inverse Probability Weighting Analysis
