# Supplementary material for: Association of Taxane Type With Patient-Reported Chemotherapy-Induced Peripheral Neuropathy Among Patients With Breast Cancer
Source: JAMA Netw Open. 2022 Nov 2;5(11):e2239788. doi: 10.1001/jamanetworkopen.2022.39788 (PMC9631104; doi:10.1001/jamanetworkopen.2022.39788)
Supplement: Supplement. — eTable 1. The EORTC QLQ-CIPN20 Scores Before and After Taxane Treatment eTable 2. Pairwise Association Between Types of Taxanes and Patient-Reported CIPN Among Patients With Breast Cancer eTable 3. Multivariate Analysis Using Semiparametric Proportional Hazards Models eFigure 1. Propensity Score Distributions According to the Types of Taxanes Used eTable 4. Overlap Propensity Score–Weighted Demographic and Clinical Characteristics of the Cohort eFigure 2. Covariate Balance Assessment in Overlap Weighting Using Maximum Pairwise Absolute Standardized Difference (ASD) eTable 5. Overlap Propensity Score–Weighted Multivariate Analysis eTable 6. Pairwise Association Between Types of Taxanes and Patient-Reported CIPN in the Inverse Probability Weighting Analysis [file jamanetwopen-e2239788-s001.pdf]

Supplemental Online Content

Mo H, Yan X, Zhao F, et al. Association of taxane type with patient-reported chemotherapy-induced peripheral neuropathy among patients with breast cancer. *JAMA Netw Open*. 2022;5(11):e2239788.
doi:10.1001/jamanetworkopen.2022.39788

eTable 1. The EORTC QLQ-CIPN20 Scores Before and After Taxane Treatment
eTable 2. Pairwise Association Between Types of Taxanes and Patient-Reported CIPN Among Patients With Breast Cancer
eTable 3. Multivariate Analysis Using Semiparametric Proportional Hazards Models
eFigure 1. Propensity Score Distributions According to the Types of Taxanes Used
eTable 4. Overlap Propensity Score–Weighted Demographic and Clinical Characteristics of the Cohort
eFigure 2. Covariate Balance Assessment in Overlap Weighting Using Maximum Pairwise Absolute Standardized Difference (ASD)
eTable 5. Overlap Propensity Score–Weighted Multivariate Analysis
eTable 6. Pairwise Association Between Types of Taxanes and Patient-Reported CIPN in the Inverse Probability Weighting Analysis

This supplemental material has been provided by the authors to give readers additional information about their work.

© 2022 Mo H et al. *JAMA Network Open*.

eTable 1. The EORTC QLQ-CIPN20 Scores Before and After Taxane Treatment

EORTC QLQ-CIPN20	**Nab-paclitaxel**	**paclitaxel**	**docetaxel**	**Between-group p value [a]**	**Between-group p value [b]**	**Between-group p value [c]**
Total Score						
Baseline	20.18±2.59	19.97±3.12	20.01±2.47	1.00	1.00	1.00
After cycle 1	22.72±4.34	20.95±3.05	21.10±3.42	0.03	0.13	1.00
After cycle 2	23.52±4.47	21.38±3.50	21.77±4.25	0.006	0.15	1.00
After cycle 3	24.76±5.56	22.24±4.64	22.26±4.71	0.0003	0.0263	1.00
After cycle 4	26.53±8.33	21.93±3.81	22.05±3.31	<.001	<.001	1.00
> 4 cycles	25.38±5.56	21.85±4.05	22.18±4.02	<.001	0.0011	1.00
Sensory scale						
Baseline	9.74±1.47	9.69±1.79	9.62±1.36	1.00	1.00	1.00
After cycle 1	11.29±2.56	10.01±1.66	10.16±1.96	0.0006	0.0377	1.00
After cycle 2	11.76±2.76	10.26±1.89	10.57±2.32	<.001	0.0119	1.00
After cycle 3	12.73±3.41	10.65±2.55	10.68±2.53	<.001	<.001	1.00
After cycle 4	13.51±4.40	10.48±2.14	10.65±1.84	<.001	<.001	1.00
> 4 cycles	12.94±3.08	10.51±2.51	10.75±2.33	<.001	<.001	1.00
Motor scale						
Baseline	8.09±1.19	7.92±1.42	8.00±1.07	1.00	1.00	1.00
After cycle 1	8.81±1.81	8.39±1.37	8.38±1.48	0.96	0.95	1.00
After cycle 2	9.05±1.91	8.51±1.64	8.55±1.78	0.59	0.92	1.00
After cycle 3	9.25±2.28	8.83±1.93	8.73±2.03	0.89	0.98	1.00
After cycle 4	10.04±3.64	8.72±1.63	8.65±1.41	0.06	0.07	1.00

© 2022 Mo H et al. *JAMA Network Open.*

> 4 cycles	9.45±2.56	8.48±1.55	8.71±1.69	0.29	0.63	1.00
Autonomic scale						
Baseline	2.35±0.62	2.36±0.58	2.39±0.57	1.00	1.00	1.00
After cycle 1	2.62±0.84	2.55±0.71	2.56±0.73	1.00	1.00	1.00
After cycle 2	2.71±0.83	2.61±0.75	2.65±0.80	1.00	1.00	1.00
After cycle 3	2.79±0.89	2.76±0.86	2.85±0.91	1.00	1.00	0.9999
After cycle 4	2.98±1.01	2.72±0.73	2.75±0.85	0.9996	0.9998	1.00
> 4 cycles	2.99±0.93	2.86±0.95	2.71±0.78	1.00	0.9812	0.9999

The above analysis used a linear mixed-effect model, and the subsequent pairwise comparisons were corrected using the Scheffé method. Score values are the observed mean ± SE.

[a] the nab-paclitaxel group versus paclitaxel group

[b] the nab-paclitaxel group versus docetaxel group

[c] the paclitaxel group versus docetaxel group

© 2022 Mo H et al. *JAMA Network Open.*

eTable 2. Pairwise Association Between Types of Taxanes and Patient-Reported CIPN Among Patients with Breast Cancer

EORTC QLQ-CIPN20		**HR (95% CI)** [a]	***P* value**	**HR (95% CI)** [b]	***P* value**	**HR (95% CI)** [c]	***P* value**
Total Score	Paclitaxel vs nab-paclitaxel	0.61 (0.51-0.74)	<.001	0.68 (0.56-0.84)	<.001	0.61 (0.49-0.75)	<.001
	Docetaxel vs nab-paclitaxel	0.65 (0.54-0.79)	<.001	0.69 (0.56-0.85)	<.001	0.66 (0.54-0.82)	<.001
	Docetaxel vs paclitaxel	1.06 (0.91-1.24)	.47	1.01 (0.86-1.19)	.90	1.09 (0.92-1.31)	.31
Sensory scale	Paclitaxel vs nab-paclitaxel	0.45 (0.38-0.54)	<.001	0.51 (0.41-0.62)	<.001	0.47 (0.38-0.58)	<.001
	Docetaxel vs nab-paclitaxel	0.49 (0.41-0.60)	<.001	0.56 (0.46-0.69)	<.001	0.54 (0.44-0.66)	<.001
	Docetaxel vs paclitaxel	1.10 (0.93-1.30)	.29	1.11 (0.93-1.31)	.26	1.14 (0.95-1.37)	.17
Motor scale	Paclitaxel vs nab-paclitaxel	0.86 (0.72-1.03)	.10	0.90 (0.74-1.11)	.33	0.76 (0.62-0.94)	.01
	Docetaxel vs nab-paclitaxel	0.71 (0.59-0.86)	<.001	0.71 (0.58-0.88)	.002	0.68 (0.55-0.85)	<.001
	Docetaxel vs paclitaxel	0.82 (0.70-0.98)	.03	0.79 (0.66-0.94)	.01	0.90 (0.74-1.08)	.26
Autonomic scale	Paclitaxel vs nab-paclitaxel	1.00 (0.83-1.21)	.98	1.07 (0.87-1.32)	.54	0.99 (0.79-1.23)	.91
	Docetaxel vs nab-paclitaxel	0.87 (0.71-1.06)	.15	0.90 (0.72-1.12)	.38	0.90 (0.72-1.12)	.35
	Docetaxel vs paclitaxel	0.87 (0.73-1.03)	.10	0.84 (0.71-1.01)	.06	0.91(0.75-1.11)	.35

[a] Univariate model only include types of taxanes.

[b] Multivariable models accounted for baseline characteristics, including patient age, ethnicity, BMI, diabetes, history of peripheral neuropathy, treatment stage and chemotherapy regimens.

[c] Model further adjusted for treatment cycles and ki67.

© 2022 Mo H et al. *JAMA Network Open.*

eTable 3. Multivariate Analysis Using Semiparametric Proportional Hazards Models

Characteristics	**Coefficient**	**95% CI [b]**		***P***
		**Lower limit**	**Upper limit**	
Age	0.00	-0.01	0.01	.90
Ethnicity (compared to the Han population)				
Other minorities	-0.10	-0.39	0.18	.48
BMI [a]	0.02	-0.13	0.16	.79
History of diabetes	0.07	-0.24	0.39	.65
History of peripheral neuropathy	0.59	-0.05	1.22	.07
Ki67% (compared to <20%)				
≥20%	-0.00	-0.20	0.19	.96
Missing	-0.23	-0.49	0.04	.09
Treatment cycles	-0.11	-0.16	-0.05	<.001
Treatment stage (compared to adjuvant patients)				
Palliative	0.28	0.06	0.51	.01
Neoadjuvant	0.07	-0.13	0.28	.47
Chemotherapy regimens (compared to monotherapy)				
Combinations	0.09	-0.07	0.24	.29
Types of taxanes (compared to nab-paclitaxel)				
Paclitaxel	-0.50	-0.72	-0.29	<.001
Docetaxel	-0.41	-0.62	-0.20	<.001

[a] Body mass index, calculated as weight in kilograms divided by height in meters squared.

[b] Confidence Interval

© 2022 Mo H et al. *JAMA Network Open.*

eFigure 1. Propensity Score Distributions According the Types of Taxanes Used

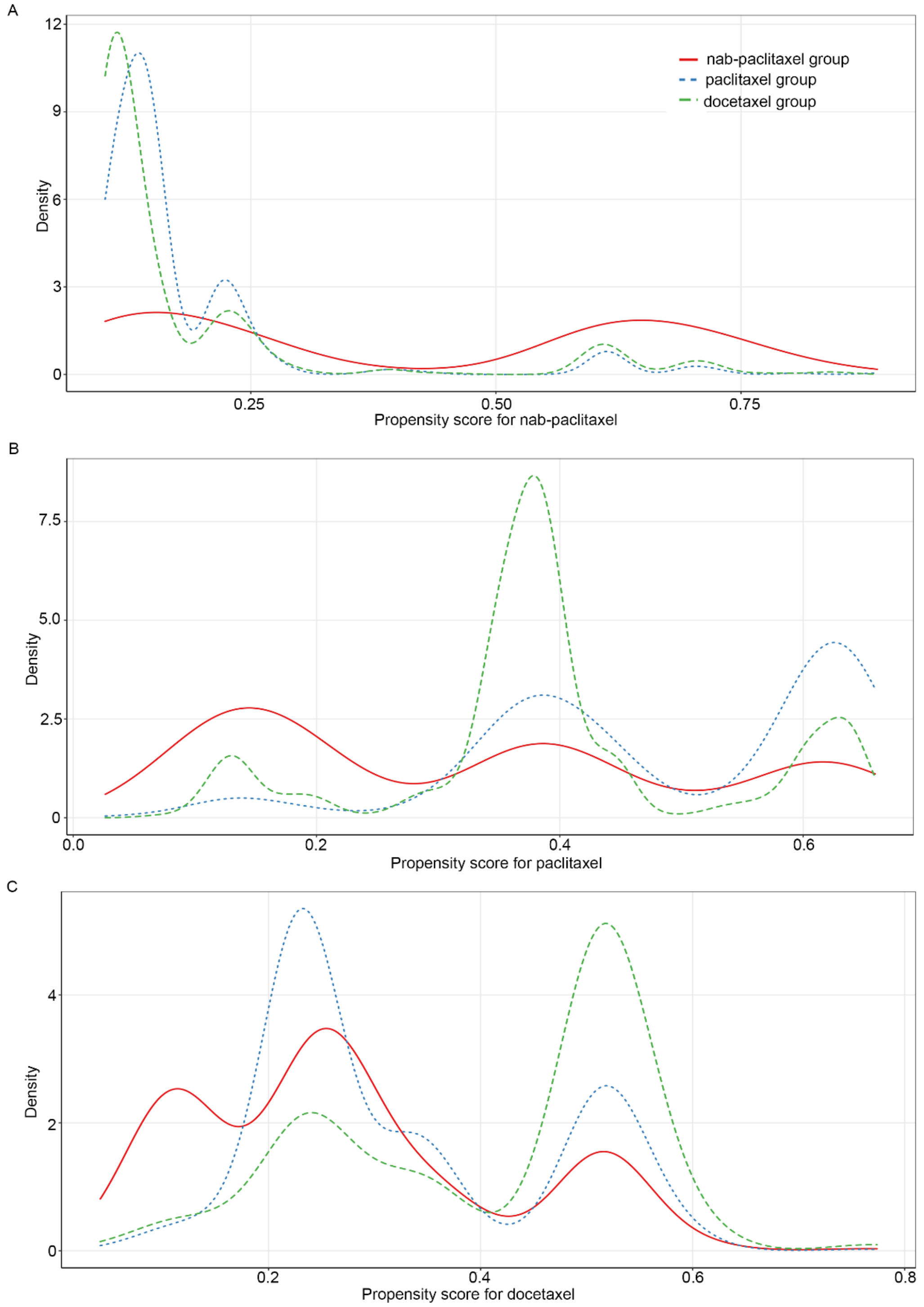

Figure legend: Density plots of estimated generalized propensity scores for nab-paclitaxel (A), paclitaxel (B) and docetaxel (C) were shown.

© 2022 Mo H et al. *JAMA Network Open.*

eTable 4. Overlap Propensity Score–Weighted Demographic and Clinical Characteristics of the Cohort[a]

	Patients, %			
Characteristics	Nab-paclitaxel	Paclitaxel	Docetaxel	*P* value
Age, mean (SD)	51.0 (11.1)	51.4 (10.5)	50.8 (9.7)	.68
Ethnicity				.98
Han	93.0	93.0	92.4	
Other minorities	7.0	7.0	7.6	
Body mass index, kg/m2 [b]	24.0	23.9	23.9	.89
Diabetes				.95
No	92.4	92.4	91.3	
Yes	7.6	7.6	8.7	
History of peripheral neuropathy				.99
No	98.1	98.0	97.8	
Yes	1.9	2.0	2.2	
Treatment stage				.99
Adjuvant	67.2	64.8	64.1	
Palliative	14.5	15.7	16.5	
Neoadjuvant	18.3	19.5	19.4	
Chemotherapy regimens				.80
Monotherapy	37.1	36.3	40.6	
Combinations [c]	62.9	63.7	59.4	

[a] Reported is either the overlap propensity score–weighted mean or proportion for each group. Comparisons between groups were performed using analysis of variance or chi-square test.

[b] Calculated as weight in kilograms divided by height in meters squared.

[c] Taxanes in combination with other chemotherapeutic drugs.

© 2022 Mo H et al. *JAMA Network Open.*

eFigure 2. Covariate Balance Assessment in Overlap Weighting Using Maximum Pairwise Absolute Standardized Difference (ASD)

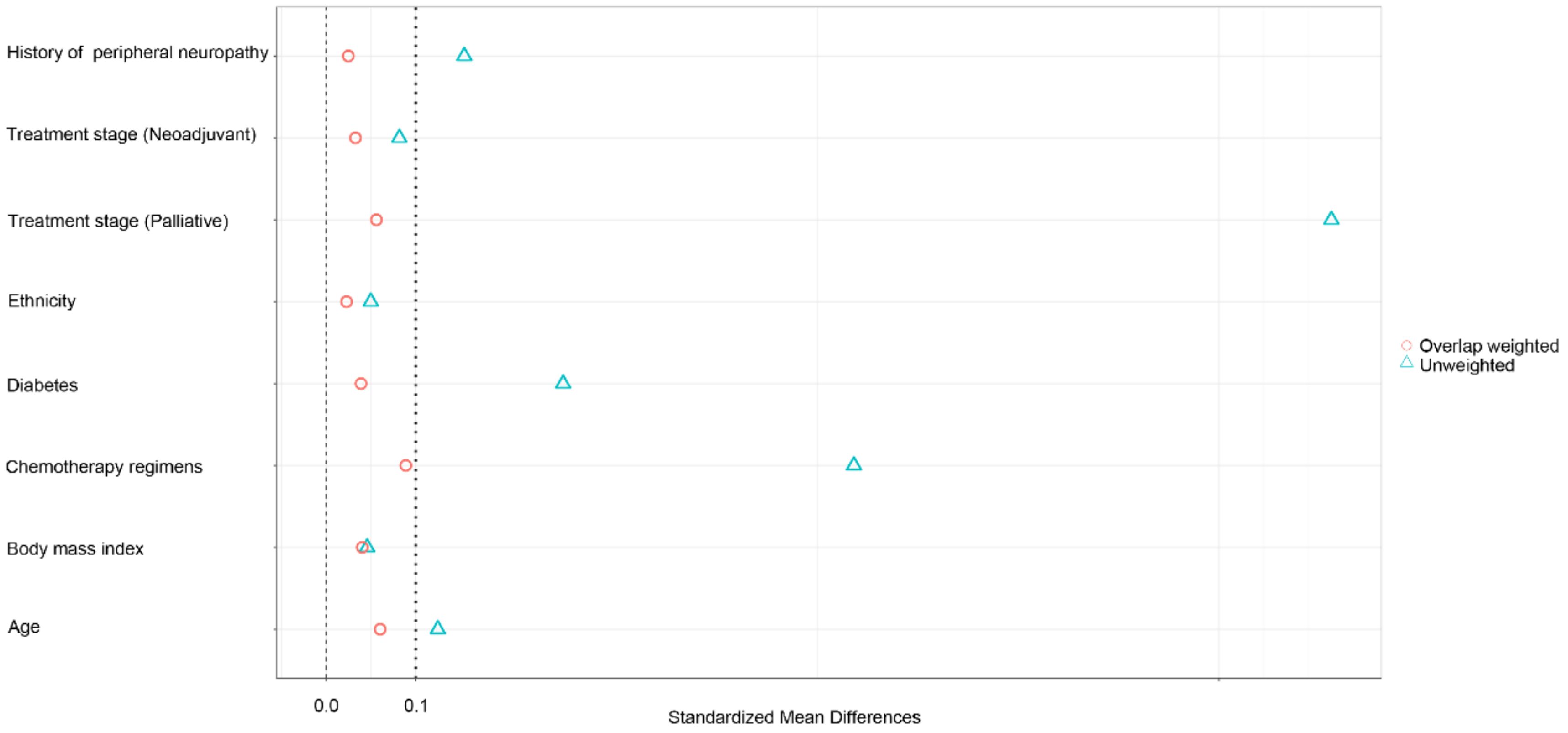

Figure legend: The covariate was considered to be balanced well when the absolute value of ASD was less than 0.1.

© 2022 Mo H et al. *JAMA Network Open.*

eTable 5. Overlap Propensity Score–Weighted Multivariate Analysis

Characteristics	**Coefficient**	**95% CI [b]**		***P***
		**Upper limit**	**Lower limit**	
Age	0.00	-0.01	0.02	.79
Ethnicity (compared to the Han population)				
Other minorities	-0.02	-0.59	0.55	.94
BMI [a]	0.04	-0.26	0.34	.80
History of diabetes	0.07	-0.51	0.65	.82
History of peripheral neuropathy	0.41	-0.82	1.64	.51
Ki67% (compared to <20%)				
≥20%	0.03	-0.40	0.47	.89
Missing	-0.26	-0.81	0.29	.35
Treatment cycles	-0.09	-0.19	0.02	.10
Treatment stage (compared to adjuvant patients)				
Palliative	0.27	-0.14	0.69	.20
Neoadjuvant	0.11	-0.28	0.50	.58
Chemotherapy regimens (compared to monotherapy)				
Combinations	0.09	-0.23	0.41	.59
Types of taxanes (compared to nab-paclitaxel)				
Paclitaxel	-0.52	-0.90	-0.13	.008
Docetaxel	-0.43	-0.80	-0.06	.02

[a] Body mass index, calculated as weight in kilograms divided by height in meters squared.
[b] Confidence Interval

© 2022 Mo H et al. *JAMA Network Open.*

eTable 6. Pairwise Association Between Types of Taxanes and Patient-Reported CIPN in the Inverse Probability Weighting Analysis

Covariates	**HR (95% CI) [a]**	***P* value**	**HR (95% CI) [b]**	***P* value**	**HR (95% CI) [c]**	***P* value**
Types of taxanes						
Paclitaxel vs nab-paclitaxel	0.68 (0.61-0.75)	<.001	0.68 (0.61-0.75)	<.001	0.60 (0.50-0.73)	<.001
Docetaxel vs nab-paclitaxel	0.67 (0.61-0.74)	<.001	0.67 (0.61-0.74)	<.001	0.66 (0.57-0.76)	<.001
Docetaxel vs paclitaxel	0.99 (0.89-1.10)	.88	0.99 (0.90-1.10)	.89	1.10 (0.98-1.23)	.12
Age	-	-	1.00 (0.99-1.00)	.94	1.00 (0.99-1.00)	.97
Ethnicity						
Other minorities vs the Han population	-	-	0.95 (0.81-1.11)	.52	0.99 (0.83-1.17)	.90
BMI	-	-	1.01 (0.92-1.10)	.90	1.00 (0.92-1.09)	.99
History of diabetes	-	-	1.05 (0.88-1.27)	.57	1.04 (0.88-1.23)	.65
History of peripheral neuropathy	-	-	1.64 (1.26-2.12)	<.001	1.63 (1.25-2.11)	<.001
Treatment stage						
Palliative vs adjuvant patients	-	-	1.24 (1.10-1.40)	<.001	1.29 (1.14-1.45)	<.001
Neoadjuvant vs adjuvant patients	-	-	1.03 (0.92-1.17)	.57	1.08 (0.96-1.22)	.18
Chemotherapy regimens						
Combinations vs monotherapy	-	-	1.14 (1.04-1.24)	.003	1.06 (0.96-1.17)	.27
Ki67% (compared to <20%)						
≥20%	-	-	-	-	1.03 (0.91-1.16)	.69
Missing	-	-	-	-	0.77 (0.66-0.90)	<.001
Treatment cycles	-	-	-	-	0.90 (0.87-0.94)	<.001

[a] Univariate model inly include types of taxanes.
[b] Multivariable models accounted for baseline characteristics, including patient age, ethnicity, BMI, diabetes, history of peripheral neuropathy, treatment stage and chemotherapy regimens.
[c] Model further adjusted for treatment cycles and ki67.
Abbreviations: HR, Harzard Ratio; CI, Confidence Interval; BMI, Body mass index, calculated as weight in kilograms divided by height in meters squared.

© 2022 Mo H et al. *JAMA Network Open.*
